# Microbial assessment of commercial pet foods marketed in the United Arab Emirates

**DOI:** 10.3389/fvets.2024.1389981

**Published:** 2024-05-24

**Authors:** Michella Hadid, Nisreen Alwan, Hani Dimassi, Maria Deghel, Sami El Khatib, Mireille Serhan, Hussein F. Hassan

**Affiliations:** ^1^Department of Natural Sciences, School of Arts and Sciences, Lebanese American University, Beirut, Lebanon; ^2^Department of Nutritional Sciences, Faculty of Health Sciences, University of Balamand, Tripoli, Lebanon; ^3^College of Health Sciences, Abu Dhabi University, Abu Dhabi, United Arab Emirates; ^4^School of Pharmacy, Lebanese American University, Byblos, Lebanon; ^5^Department of Food Sciences and Technology, School of Arts and Sciences, Lebanese International University, Bekaa, Lebanon; ^6^Department of Biological Sciences, School of Arts and Sciences, Lebanese International University, Bekaa, Lebanon; ^7^Center for Applied Mathematics and Bioinformatics (CAMB) at Gulf University for Science and Technology, Hawally, Kuwait

**Keywords:** pet food, bacteria, microbiological quality, safety, UAE

## Abstract

Examining the microbiological characteristics of pet food is imperative to safeguard the health and well-being of companion animals, pet owners, and the surrounding environment. Domestic animals, known for carrying harmful microorganisms, pose a significant health risk, especially in close proximity to people and children. Notably, no studies have previously investigated pet food quality in the Gulf Cooperation Council countries, in particular, the United Arab Emirates (UAE). This study examined the microbiological quality of all stock keeping units (SKUs) of pet foods marketed in UAE (*n* = 118). Parameters assessed include Total Aerobic Microbial Count (TAMC), *Enterobacteriaceae*, Total Yeast and Mold Count (TYMC), *Salmonella*, *Listeria monocytogenes*, and *Clostridium* species. Among the 118 samples, 33 (28%) exceeded the acceptable TAMC limit of 10^6^ CFU/g, highlighting significant variations based on manufacturers and ingredients. Eight samples (7%) surpassed the maximum *Enterobacteriaceae* limit of 3 × 10^2^ CFU/g. TYMC levels exhibited variation, with 33 (28%) exceeding the limit of 10^4^ CFU/g. *L. monocytogenes* was identified in 44 (37%) of the samples, while *Salmonella* was not detected. *Clostridium* contamination was observed in 28 (24%) of the samples. Statistical analyses revealed associations between pet food characteristics and microbial quality, underscoring the imperative for international standards to ensure the safety of pet food. These findings carry significant implications for pet owners, regulatory bodies, and the pet food industry, emphasizing the need for ongoing efforts to enhance the overall quality and safety of pet food products.

## Highlights

- Dry pet food items exhibited greater levels of contamination in comparison to canned ones.
- (28%) samples had a total aerobic microbial count exceeding 10^6^ CFU/g.
- 8 (7%) samples surpassed the maximum limit of 3×10^2^ CFU/g for *Enterobacteriacea.*
- 33 (28%) samples had a contamination level of total yeasts and molds above 10^4^ CFU/g.
- *Listeria monocytogenes* was present in 44 (37%) of the stock keeping units while *Salmonella* was not detected.
- *Clostridium* was detected in 28 (24%) of samples.

## 1 Introduction

In contemporary times, the trend of pet ownership, notably centered around cats and dogs, is witnessing a significant surge worldwide. Statistics reveal that roughly ∼80 million households across Europe (1) and approximately 60% of households in the United States (2) embrace at least one pet. This upswing was particularly noticeable during the COVID-19 pandemic, highlighting the invaluable role of pets as companions, offering comfort and aiding individuals in managing stress, ultimately contributing to improved health (3). As the population of pets continues to rise, the pet food market is undergoing dynamic evolution. Since the 1940s, the manufacturing of pet food in Europe and the United States has been based on animal feeds initially intended for livestock, leading to the establishment of numerous pet food manufacturing facilities in most developed nations (4).

The safety of pet food is a paramount concern, not only for the well-being of pets but also for pet owners and environmental conservation. Apart from the nutritional value, ensuring the microbiological safety of pet food is a crucial criterion in delivering safe and healthy sustenance (5). Studies indicate that approximately 90% of pet owners in both the United States and Australia prefer commercial pet food due to its perceived convenience in meeting pets’ nutritional requirements and its cost-effectiveness compared to homemade alternatives (6, 7). Notably, there has been a discernible shift in feeding practices between 2008 and 2018, witnessing an increased inclination toward homemade or non-traditional diets. This shift is driven by perceptions of affordability, taste preferences, nutritional concerns, and apprehensions about added chemicals and preservatives (6–8). Additionally, both canned and dry commercial pet food may degrade in quality over time after purchase, despite initial assurances of safety and health (9). Various hazards lurk within pet food, posing potential health risks to pets. These hazards encompass chemical threats like cyanuric acid (10) and physical hazards such as metal and other hard particles (11). After processing, one of the pivotal aspects of ensuring pet food safety revolves around its microbiological quality, particularly regarding the presence or absence of zoonotic agents. Prior research has identified pathogens such as *Enterobacteriaceae*, *Salmonella*, *L. monocytogenes, Clostridium* species, and molds in both dry and canned pet food products (12–16).

Microorganisms present in pet food not only pose health risks to pets but also to their owners who share close bonds with them. Contaminated pet food has been implicated in human illnesses through various means, including direct interaction or indirect contact between humans and items contaminated with pet food. Moreover, some pets may carry diseases without displaying any symptoms themselves (17). To ensure the safety of pet food, regulatory bodies such as the Food and Drug Administration (FDA), the United States Department of Agriculture, and State feed agencies have established specific guidelines and regulations governing the manufacturing and labeling of pet food. Kukier et al. (18) emphasized the importance of microbiological quality in livestock feed, setting a maximum allowable total aerobic microbial count (TAMC) at 10^6^ CFU/g.

According to EU regulations No 142/2011 (19) processed pet food samples, excluding canned pet food, exceeding 3 × 10^2^ CFU/g of *Enterobacteriaceae* are deemed unsatisfactory for microbial hygiene. Notably, there are no explicit regulations specifying the limit of *L. monocytogenes monocytogenes* species in pet food (20). It is presumed that *L. monocytogenes* species should adhere to the standards established for human foods, requiring their absence in 25 g of the feed (21). Additionally, EU regulations (19) dictate that *Salmonella* must be entirely absent in 25 g of pet food, and the total count of yeasts and molds should not exceed >10^4^ CFU/g (22). These stringent measures collectively aim to uphold the microbial safety and quality of pet food products.

In the United Arab Emirates (UAE) and the broader Middle East and North Africa (MENA) region, there has been a recent surge in pet ownership. Nevertheless, comprehensive data remains scarce. To the best of our knowledge, no studies have been undertaken on the microbiological safety of pet food in the Gulf Cooperation Council, including the UAE. Furthermore, only one study has been conducted in the Arab region, specifically in Lebanon (16); however, it is important to emphasize the regional specificity in assessing the microbiological safety of pet food products. The United Arab Emirates (UAE) and Lebanon are distinct regions with differences in environmental conditions, manufacturing practices, transportation logistics, and regulatory frameworks. These variations can significantly impact the microbial composition and safety of pet food products. Furthermore, the UAE is a rapidly growing market with unique socio-economic factors and cultural influences that may affect pet food production, distribution, and storage. Therefore, it is crucial to evaluate the microbiological safety of pet food specifically in the UAE to provide region-specific data for consumers, regulatory authorities, and pet food manufacturers. This study addresses the gap in current knowledge by conducting a comprehensive investigation into the microbiological safety of pet food in the UAE, contributing essential data to enhance awareness and inform regulatory measures.

## 2 Materials and methods

### 2.1 Sampling plan

To assess a diverse array of commercially available dog and cat foods across the United Arab Emirates (UAE), the market got screened and 118 stock keeping units (SKUs; 91 canned products and 27 dry products) were identified and gathered from various pet food stores and supermarkets throughout the country in 2023. Table 1 provides a comprehensive breakdown of the SKU details, encompassing sample type, pet type, pet age, protein source, grain or grain-free composition, and country of origin. All collected samples were maintained in their original packaging and remained unopened until analysis. Each sample underwent two separate tests for Salmonella and *L. monocytogenes* spp. detection, enumeration of Enterobacteriaceae spp., detection of Clostridium spp. and evaluation of total aerobic microbial count (TAMC) and total yeasts and molds count (TYMC).

**Table 1:** Characteristics of dry and canned pet food samples (*n* = 118).

	**N**	**%**
**Sample type**		
Dry	27	22.9%
Canned	91	77.1%
Total	118	100.0%
**Pet type**		
Cats	94	79.7%
Dogs	24	20.3%
Total	118	100.0%
**Pet’s Age**		
Adult	109	92.4%
Puppy or Kitten	7	5.9%
Both	2	1.7%
Total	118	100.0%
**Grain**		
Present	111	94.1%
Grain free	7	5.9%
Total	118	100.0%
**Source of Protein combined**		
Poultry	78	66.1%
Fish	56	47.5%
Beef	30	25.4%
Liver	17	14.4%
Turkey	13	11.0%
Lamb	3	2.5%
Duck	2	1.7%
Wild game	1	0.8%
Rabbit	1	0.8%
**Country**		
Thailand	43	36.4%
USA	21	17.8%
Hungary	18	15.3%
France	17	14.4%
Others	19	16.1%

### 2.2 Microbiological analysis

The processing and dilution of the samples adhered to the standard ISO 6887–1:2017b − 5 protocol (23). A 25 g portion from each sample was combined with nine times its volume (∼225 mL) of buffered peptone water (Biolife, Italy) and homogenized for 1–2 min using a stomacher (BagMixer 400 W, interscience, France). Subsequently, a 10-fold serial dilution in 0.1% (v/v) peptone water was prepared. A volume of 0.1 mL from the mother solution (MS) and each diluted mixture was separately transferred onto petri dishes. The detection of certain microorganisms (Salmonella, *L. monocytogenes* and Clostridium species) and enumeration of others (TAMC, TYMC, and Enterobacteriaceae species) occurred following specific incubation periods and temperatures. The examination of pet food samples complied with established standards governing the microbiology of food and feeding materials (24).

Total aerobic microbial count (TAMC): Each sample underwent dilution stages of 10^−1^, 10^−2^, 10^−3^, 10^−4^, 10^−5^, and 10^−6^. A 0.1 mL aliquot from each dilution was spread onto plate count agar (PCA) agar (Biolife, Italy) for incubation at 37°C ± 1°C for 42 h. All developed colonies on the plates were enumerated.

*Enterobacteriaceae* enumeration: The samples underwent dilution at the following ratios: 10^−1^, 10^−2^, 10^−3^, 10^−4^, 10^−5^, and 10^−6^. Subsequently, 0.1 mL from each dilution was plated on Violet Red Bile Glucose (VRBG) agar (Biolife, Italy) for incubation at 37°C ± 1°C for 48 h. Colonies displaying red coloration with red-pink halos on the plates were considered presumptive *Enterobacteriaceae* species and were enumerated.

Yeasts and molds enumeration: Samples underwent dilutions at 10^−1^, 10^−2^, 10^−3^, 10^−4^, 10^−5^, and 10^−6^ and 0.1 mL from each dilution was spread onto Sabouraud agar (Himedia, India) for incubation at 25°C ± 1°C for 5 days. Colonies observed on the plates were suspected to be yeasts and molds.

*Salmonella* detection: Isolation of *Salmonella* spp. involved a two-step enrichment process. After incubating samples at 37°C ± 1°C for 24 h in Buffered Peptone Water, inoculations were made into Rappaport-Vassiliadis RVS broth and tetrathionate broth (Biolife, Italy). Following incubation at 42°C ± 1°C for 24 h, plating onto XLD and *Salmonella Shigella* Agar (Biolife, Italy) occurred and subsequent incubation at 37°C for 24 h. The appearance of black colonies post-incubation indicates presumptive *Salmonella* species.

*L. monocytogenes* detection: Enrichment involved incubation at 37°C ± 1°C for 24 h in Buffered Peptone Water, followed by inoculation into Frazer broth and incubation at 42°C ± 1°C for 24 h. Smears from the broth were spread onto Palcam agar (Biolife, Italy), and the appearance of black colonies post-incubation at 37°C ± 1°C for 24 h implies the presence of presumptive *L. monocytogenes* species.

*Clostridium* detection: The samples underwent dilution at the following ratios: 10^−1^, 10^−2^, 10^−3^, 10^−4^, 10^−5^, and 10^−6^. A 0.1 mL volume of each dilution was plated onto Blood Agar Base (Biolife, Italy) supplemented with 5–7% sterile defibrinated horse blood. These plates were then transferred into an anaerobic jar to create anaerobic conditions using OXOID AnaeroGen, Anaerobic generator sachets in 2.5 L jar. Incubation was carried out at 37°C ± 1°C for 48 h. Colonies generated by *Clostridium* species frequently exhibit distinct characteristics, including irregular edges, central protrusion, and a visual aspect reminiscent of “ground glass.”

Following ISO 7218 guidelines (24), the presence and quantity of microorganisms were analyzed, expressing microbial counts as the logarithm of colony-forming units per gram of sample.

Results, including concentrations of Total Aerobic Microbial Count (TAMC), Enterobacteriaceae, and Total Yeast and Mold Count (TYMC), were systematically categorized based on their proximity to the acceptable limit. Specifically, they were classified as either falling below the acceptable limit, signifying acceptability, or exceeding it, indicating unacceptability. The set limits were 106 for TAMC, 104 for TYMC, and 300 CFU/g (colony-forming units per gram) for Enterobacteriaceae.

### 2.3 Statistical analysis

Information regarding pet food products and laboratory analysis results were encoded and entered into SPSS V26 for subsequent analysis. Microorganism concentrations, including TAMC, Enterobacteriaceae, and TYMC, were regrouped and subjected to bivariate analysis using Pearson Chi-square, or fisher exact test when expected cell sizes were small, to assess the effect of pet food characteristics. Statistical significance was denoted by a *p*-value <0.05.

## 3 Results and discussion

For TAMC, TYMC, and *Enterobacteriaceae*, concentrations ranged from below the quantification limit to exceeding 3 × 10^7^ CFU/g. Within the TAMC and TYMC categories, 33 samples (28%) exhibited contamination levels surpassing the established limit, while 85 samples (72%) recorded contamination levels below it. Conversely, *Enterobacteriaceae* was detected in 8 samples (7%).

*Salmonella* and *L. monocytogenes* results were stratified based on the presence or absence of these bacteria, complemented by an additional evaluation for *Clostridium*. *L. monocytogenes* species were identified in 44 samples (37%), whereas S*almonella* species were absent in all samples. Furthermore, 28 (24%) samples were found to be contaminated with *Clostridium*, with contamination levels ranging from below the quantification limit to 3 × 10^3^ CFU/g. This meticulous categorization ensures a thorough assessment of microbial quality, verifying compliance with specified limits, and offering valuable insights into potential food safety concerns.

Tables 2–7 present statistical findings pertaining to the microbiological quality of both dry and canned pet food. A noteworthy correlation was observed between the nature of pet food (dry/canned) and the contamination level of TAMC at a significance level of *p* < 0.05. Additionally, a significant disparity was identified in the contamination with *L. monocytogenes* species based on the type of pet (dog/cat). Furthermore, a significant distinction emerged between the country of origin and the contamination levels of both TAMC and *Clostridium* species.

**Table 2:** Counts for different microorganisms in the 118 samples.

No	TAMC	TYMC	*Clostridium* spp.	*Enterobacteriacea* spp.	*Salmonella* spp.	*Listeria* spp.
**cfu/g**						
1	1,550,000	5,000	175	300	-	-
2	25,000	55,000,000	10	5	-	+
3	205,000	35	ND	6,000,000	-	-
4	1,050	2,350,000	ND	ND	-	-
5	1,700,000	5,000	10	ND	-	-
6	200	5	ND	ND	-	-
7	291,000,000	50,000	ND	ND	-	-
8	27,000	30	ND	ND	-	-
9	9,200,000	195	ND	ND	-	+
10	1,830,000	225,000	35	12,000,000	-	+
11	4,900	300	ND	ND	-	-
12	5,200	395	ND	ND	-	-
13	7,700	150	ND	ND	-	+
14	7,300,000	200	ND	ND	-	+
15	17,500	70	ND	ND	-	+
16	167,000,000	130	5	ND	-	+
17	1,100,000	25,500	ND	ND	-	+
18	2,000,000	45,000	ND	ND	-	+
19	340,000,000	ND	200	ND	-	+
20	38,000,000	500	ND	ND	-	-
21	10,000,000	700,000	ND	ND	-	-
22	1,000,000	25	ND	ND	-	+
23	55	500	ND	ND	-	-
24	1,500	ND	ND	ND	-	+
25	ND	16,000,000	ND	ND	-	-
26	350	500	ND	ND	-	+
27	50	450,000	ND	ND	-	+
28	190	550	ND	ND	-	+
29	50	20,000	ND	ND	-	-
30	10	15,000	ND	ND	-	+
31	100	5	ND	ND	-	+
32	10	15	ND	ND	-	+
33	65	300	ND	ND	-	-
34	5,850	5	ND	ND	-	+
35	1,200	15	ND	ND	-	-
36	63,000	500	ND	ND	-	-
37	100	50	ND	ND	-	-
38	5	2,500	ND	ND	-	-
39	2,500	250	ND	ND	-	-
40	5	50	ND	ND	-	-
41	284,000,000	10	415	ND	-	-
42	3,950,000	10	3,680	ND	-	-
43	15,650	200	ND	ND	-	-
44	1,500	200	ND	ND	-	-
45	50,000	20	ND	1,000	-	-
46	350,000	15	ND	ND	-	-
47	4,400	30	ND	ND	-	-
48	1,385,000	1,500	ND	ND	-	-
49	5,000	155,000	ND	ND	-	-
50	14,000	10	ND	ND	-	-
51	150	25	ND	ND	-	-
52	15	30,000	ND	300	-	-
53	16,950	20	ND	ND	-	-
54	8,150,000	100	ND	ND	-	-
55	46,000	45	ND	ND	-	-
56	16,000	95	ND	ND	-	-
57	800	200	10	ND	-	-
58	4,800,000	12,500	230	ND	-	-
59	1,510,000	295	ND	ND	-	+
60	425	11,500	ND	ND	-	+
61	1,500	1,500	ND	ND	-	+
62	137,000	2,450	ND	ND	-	+
63	85	22,500	ND	ND	-	+
64	68,000,000	10,500	ND	ND	-	+
65	600,000	50	ND	ND	-	+
66	9,000	45,000	ND	ND	-	+
67	2,500,000	100	ND	ND	-	-
68	2000	4,350	ND	24,500	-	+
69	3,000	3,500	ND	50,000	-	+
70	7,700,000	20,000	ND	ND	-	+
71	5,500	200	ND	ND	-	-
72	9,000	2,400,000	ND	ND	-	+
73	8,500,000	550,000	ND	ND	-	+
74	14,500	1,500,000	ND	ND	-	-
75	210	2,500	ND	ND	-	-
76	1840	11,000,000	ND	ND	-	+
77	350	60	ND	ND	-	-
78	380,000,000	11,500,000	ND	ND	-	-
79	4,350	270,000	ND	ND	-	+
80	750	3,050	ND	ND	-	+
81	1,900,000	50	ND	ND	-	+
82	450	50	ND	ND	-	+
83	165,000	75	55	ND	-	-
84	18,000	8,500	20	ND	-	-
85	115	5	85	ND	-	+
86	200	55,000,000	5	500,000	-	-
87	10	15	ND	ND	-	-
88	5	1,300	ND	ND	-	-
89	145	1,100	5	ND	-	-
90	10	55,000	10	ND	-	+
91	900,000	3,000	5	ND	-	+
92	225,000	100	10	ND	-	-
93	3,800	5,000	5	ND	-	-
94	950	50	5	ND	-	-
95	18,000,000	50,000	10	5	-	-
96	1,350	50	ND	ND	-	+
97	96,000	5,000	ND	ND	-	+
98	50	15,000	ND	ND	-	+
99	9,500,000	ND	5	ND	-	-
100	17,000	45	ND	ND	-	-
101	240,000,000	5,000	ND	ND	-	-
102	17,000,000	5	ND	ND	-	+
103	700,000	4,000	ND	ND	-	-
104	5,050	2,650,000	ND	ND	-	-
105	240	250,000	ND	ND	-	-
106	230,000,000	40	ND	ND	-	-
107	2,700	3,000	20	ND	-	-
108	205,500	10,000	ND	ND	-	-
109	545	200	5	ND	-	-
110	TNTC	40	ND	ND	-	-
111	140,000,000	2000	ND	ND	-	-
112	365	20	40	ND	-	-
113	71,000	17,500	ND	ND	-	-
114	5,650	75	100	ND	-	-
115	440,000	150	5	ND	-	-
116	9,500,000	450,000	ND	ND	-	-
117	900	50	5	1,500,000	-	-
118	150,000,000	2000	ND	5,000	-	-

ND, not detected.

**Table 3:** Microbiological results of total aerobic microbial count (TAMC) in dry and canned pet food.

TAMC	Acceptable/below limit 10^6^		Not acceptable/above 10^6^		*p*-value
	N	%	N	%	
**Sample Type**					
Dry	15	55.60%	12	44.40%	
Canned	70	76.90%	21	23.10%	**0.030**
**Pet type**					
Cats	70	74.50%	24	25.50%	
Dogs	15	62.50%	9	37.50%	0.244
**Pet’s Age**					
Adult	80	73.40%	29	26.60%	
Puppy or Kitten	4	57.10%	3	42.90%	
Both	1	50.00%	1	50.00%	0.334
**Grain**					
Present	67	76.10%	21	23.90%	
Grain free	18	60.00%	12	40.00%	0.095
**Food Source**					
Poultry: Present	59	75.60%	19	24.40%	
Absent	26	65.00%	14	35.00%	0.223
Fish: Present	41	73.20%	15	26.80%	
Absent	44	71.00%	18	29.00%	0.786
Beef: Present	19	63.30%	11	36.70%	
Absent	66	75.00%	22	25.00%	0.219
Liver: Present	13	76.50%	4	23.50%	
Absent	72	71.30%	29	28.70%	0.777
Turkey: Present	9	69.20%	4	30.80%	
Absent	76	72.40%	29	27.60%	0.755
Lamb	--		--		--
Duck	--		--		--
Wild game	--		--		--
Rabbit	--		--		--
**Country**					
France	11	64.70%	6	35.30%	
Hungary	7	38.90%	11	61.10%	
Thailand	36	83.70%	7	16.30%	
USA	19	90.50%	2	9.50%	
Others	12	63.20%	7	36.80%	**0.002**

Significance level: *p* = 0.05.

**Table 4:** Microbiological results of presumptive *Enterobacteriaceae* species in canned and dry pet food.

Enterobacteriaceae	Acceptable/below limit		Not acceptable/above limit 300		*p*-value
	N	%	N	%	
**Sample Type**					
Dry	23	85.20%	4	14.80%	
Canned	87	95.60%	4	4.40%	0.079
**Pet Type**					
Cats	88	93.60%	6	6.40%	
Dogs	22	91.70%	2	8.30%	0.664
**Pet’s Age**					
Adult	101	92.70%	8	7.30%	
Puppy or Kitten	7	100.00%	0	0.00%	
Both	2	100.00%	0	0.00%	0.999
**Grain**					
Present	82	93.20%	6	6.80%	
Grain free	28	93.30%	2	6.70%	0.999
**Food Source**					
Poultry: Present	74	94.90%	4	5.10%	
Absent	36	90.00%	4	10.00%	0.441
Fish: Present	52	92.90%	4	7.10%	
Absent	58	93.50%	4	6.50%	0.999
Beef: Present	28	93.30%	2	6.70%	
Absent	82	93.20%	6	6.80%	0.999
Liver: Present	16	94.10%	1	5.90%	
Absent	94	93.10%	7	6.90%	0.999
Turkey: Present	11	84.60%	2	15.40%	
Absent	99	94.30%	6	5.70%	0.214
Lamb					
Duck					
Wild game					
Rabbit					
**Country**					
France	15	88.20%	2	11.80%	
Hungary	16	88.90%	2	11.10%	
Thailand	41	95.30%	2	4.70%	
USA	20	95.20%	1	4.80%	
Others	18	94.70%	1	5.30%	0.748

Significance level: *p* = 0.05.

**Table 5:** Microbiological results of total yeasts and molds count (TYMC) in canned and dry pet food.

TYMC	Acceptable/below limit 10^4^		Not acceptable/Above limit 10^4^		*p*-value
	N	%	N	%	
**Sample Type**					
Dry	18	66.70%	9	33.30%	
Canned	67	73.60%	24	26.40%	0.479
**Pet Type**					
Cats	67	71.30%	27	28.70%	
Dogs	18	75.00%	6	25.00%	0.717
**Pet’s Age**					
Adult	79	72.50%	30	27.50%	
Puppy or Kitten	4	57.10%	3	42.90%	
Both	2	100.00%	0	0.00%	0.445
**Grain**					
Present	62	70.50%	26	29.50%	
Grain free	23	76.70%	7	23.30%	0.971
**Food Source**					
Poultry: Present	56	71.80%	22	28.20%	
Absent	29	72.50%	11	27.50%	0.936
Fish: Present	43	76.80%	13	23.20%	
Absent	42	67.70%	20	32.30%	0.274
Beef: Present	20	66.70%	10	33.30%	
Absent	65	73.90%	23	26.10%	0.448
Liver: Present	13	76.50%	4	23.50%	
Absent	72	71.30%	29	28.70%	0.777
Turkey: Present	10	76.90%	3	23.10%	
Absent	75	71.40%	30	28.60%	0.999
Lamb					
Duck					
Wild game					
Rabbit					
**Country**					
France	11	64.70%	6	35.30%	
Hungary	9	50.00%	9	50.00%	
Thailand	33	76.70%	10	23.30%	
USA	16	76.20%	5	23.80%	
Others	16	84.20%	3	15.80%	0.144

**Table 6:** Microbiological results of presumptive *Listeria* species in canned and dry pet food.

	Not acceptable/positive for *Listeria*		Acceptable/negative for *Listeria*		*p*-value
	N	%	N	%	
**Sample Type**					
Dry	11	40.70%	16	59.30%	
Canned	33	36.30%	58	63.70%	0.673
**Pet type**					
Cats	40	42.60%	54	57.40%	
Dogs	4	16.70%	20	83.30%	**0.019**
**Pet’s Age**					
Adult	38	34.90%	71	65.10%	
Puppy or Kitten	4	57.10%	3	42.90%	
Both	2	100.00%	0	0.00%	0.073
**Grain**					
Present	31	35.20%	57	64.80%	
Grain free	13	43.30%	17	56.70%	0.999
**Food Source**					
Poultry: Present	29	37.20%	49	62.80%	
Absent	15	37.50%	25	62.50%	0.973
Fish: Present	23	41.10%	33	58.90%	
Absent	21	33.90%	41	66.10%	0.419
Beef: Present	8	26.70%	22	73.30%	
Absent	36	40.90%	52	59.10%	0.164
Liver: Present	7	41.20%	10	58.80%	
Absent	37	36.60%	64	63.40%	0.720
Turkey: Present	7	53.80%	6	46.20%	
Absent	37	35.20%	68	64.80%	0.230
Lamb					
Duck					
Wild game					
Rabbit					
**Country**					
France	8	47.10%	9	52.90%	
Hungary	11	61.10%	7	38.90%	
Thailand	14	32.60%	29	67.40%	
USA	6	28.60%	15	71.40%	
Others	5	26.30%	14	73.70%	0.129

Significance level: *p* = 0.05.

**Table 7:** Microbiological results of presumptive *Clostridium* species in canned and dry pet food.

	Not contaminated		Contaminated		*p*-value
	N	%	N	%	
**Sample Type**					
Dry	18	66.70%	9	33.30%	
Canned	72	79.10%	19	20.90%	0.182
**Pet type**					
Cats	74	78.70%	20	21.30%	
Dogs	16	66.70%	8	33.30%	0.215
**Pet’s Age**					
Adult	84	77.10%	25	22.90%	
Puppy or Kitten	4	57.10%	3	42.90%	
Both	2	100.00%	0	0.00%	0.390
**Grain**					
Present	65	73.90%	23	26.10%	
Grain free	25	83.30%	5	16.70%	0.999
**Food Source**					
Poultry: Present	57	73.10%	21	26.90%	
Absent	33	82.50%	7	17.50%	0.361
Fish: Present	42	75.00%	14	25.00%	
Absent	48	77.40%	14	22.60%	0.758
Beef: Present	25	83.30%	5	16.70%	
Absent	65	73.90%	23	26.10%	0.292
Liver: Present	15	88.20%	2	11.80%	
Absent	75	74.30%	26	25.70%	0.210
Turkey: Present	12	92.30%	1	7.70%	
Absent	78	74.30%	27	25.70%	0.150
Lamb					
Duck					
Wild game					
Rabbit					
**Country**					
France	17	100.00%	0	0.00%	
Hungary	13	72.20%	5	27.80%	
Thailand	28	65.10%	15	34.90%	
USA	18	85.70%	3	14.30%	
Others	14	73.70%	5	26.30%	**0.029**

Significance level: *p* = 0.05.

The range of potential biological hazards present in pet food, capable of causing illnesses in animals if not vigilantly monitored, encompasses *Enterobacteriaceae*, *Clostridium*, yeasts and molds, *Salmonella*, and *L. monocytogenes* (25). As emphasized by Kim et al. (26), the global assurance of food safety and the minimization of food loss necessitate a comprehensive monitoring approach, particularly focusing on biological hazards, throughout the entire food supply chain. For instance, the microbiological quality of meat is contingent on various factors such as the animal’s physiological condition during slaughter, processing methods, as well as storage and transportation conditions (27).

This investigation underscores that both canned and dry pet food products have the potential to harbor food-borne pathogens, including *Enterobacteriaceae*, *Clostridium*, *L. monocytogenes*, and fungi. Consequently, pet owners are strongly advised to exercise caution and adopt stringent safety measures when handling pet food. This precautionary approach is imperative to mitigate the risks associated with these contaminants and safeguard the health of companion animals.

### 3.1 Total aerobic microbial count

The proliferation of microorganisms in food can lead to undesirable changes, resulting in spoilage and posing potential health risks to consumers. Although there are no stringent regulations specifying maximum permissible levels of bacterial and fungal contamination in pet food (20, 28), Kukier et al. (18) recommend that TAMC should not exceed 10^6^ CFU/g.

Our research reveals a wide range of TAMC in pet food samples, ranging from below the quantification limit (BQL) to values exceeding 3 × 10^7^ CFU/g. Significant variability exists among samples from different manufacturers and among samples from the same manufacturer with distinct main ingredients or intended for different pet groups (specific data not presented). Notably, 28% (33 samples) exhibited TAMC contamination levels exceeding 10^6^ CFU/g. This contrasts with findings from other studies, such as Holda et al. (12) reporting a 75% contamination rate in dry foods with lower ranges (1.0 × 10^1^ to 2.7 × 10^2^ CFU/g) and Kazimierska et al. (28) and Serhan et al. (16) showing lower percentages in European and Lebanese markets, respectively.

The microbiological quality of animal feed, including pet foods, is often compromised by unhygienic conditions during preparation, distribution, and storage, posing health risks to both humans and pets (29). Factors influencing microorganism multiplication during storage include pH, water, light, time, nutrients, inhibitors, and oxygen (30, 31). It is imperative to translate these findings into actionable regulatory measures for the industry, including comprehensive labeling requirements for pet food, adherence to Generally Recognized as Safe (GRAS) standards set by the Association of American Feed Controls Officials (AAFCO) or approved food additives, and strict adherence to good manufacturing practices (GMP), ideally with AAFCO affiliation (32). Rigorous record-keeping and proper storage practices are vital components of GMP, ensuring the integrity of pet food throughout production and distribution. Implementing HACCP is essential to minimize hazards, particularly microbial contamination, through thermal treatments to eliminate pathogens and proactive risk identification and mitigation throughout the production process.

Dry pet food showed a higher prevalence of samples with TAMC levels deemed unacceptable (*p*-value = 0.030), indicating a significant variance in susceptibility to microbial contamination compared to canned pet foods. This aligns with findings from Lebanon (16) and underscores the importance of considering pet food type for microbiological safety. The elevated risk associated with dry pet food may necessitate stricter quality control measures due to its composition and storage requirements. The primary factors contributing to pathogenic contamination include deviations from GMP and potential for cross-contamination from various sources (33). Furthermore, dry pet products show an increased susceptibility to bacterial contamination following heat treatment, unlike canned alternatives. Canned foods are generally considered safer against biological hazards like bacteria and parasites due to the typical sterilization of cans (34). Extended storage of opened dry foods, driven by their substantial feed content, may contribute to re-contamination, whereas cans and canned foods are typically consumed in a single use. The challenging weather conditions in the United Arab Emirates (UAE), characterized by high temperatures, pose obstacles in maintaining optimal storage conditions for dry pet food. The region’s elevated ambient temperatures and humidity fluctuations create an accelerate microbial growth, further compromising food quality. Manufacturers must therefore implement rigorous quality control measures, adhere to recommended storage conditions, and explore packaging solutions tailored to mitigate contamination risks in the UAE’s climate.

Pet foods intended for cats showed a slightly higher adherence to acceptable TAMC levels compared to pet foods intended for dogs. Samples from puppies or kitten samples show a slightly lower compliance rate than adult pet samples. However, there were no statistical significance for both categories (*p*-value = 0.244 and *p*-value = 0.334 respectively).

TAMC levels in grain-free foods (40.00%) show a higher percentage of contamination compared to grain-containing foods (23.90%). Nonetheless, with a p-value of 0.095, the observed difference suggests a lack of statistical significance between the two. The non-significant p-value implies that the observed disparity in microbial counts between samples with and without grains could be due to random chance. However, it is crucial to acknowledge the trend indicating a higher TAMC in samples containing grains. Our research reveals contrasting findings compared to the study conducted in Lebanon by Serhan et al. (16), which illustrated that dry samples containing grains showed higher TAMC contamination than grain-free samples.

In scrutinizing individual ingredients within pet food formulations, such as poultry, fish, beef, liver, and turkey, discernible variations in microbial quality become apparent. Notably, poultry-based pet foods demonstrate a significantly higher proportion of samples adhering to acceptable TAMC levels compared to formulations devoid of poultry. Similar patterns are observed for fish and liver components, where their inclusion correlates with elevated proportions of samples meeting acceptable TAMC criteria. These findings emphasize the influence of ingredient composition on the microbial quality of pet food. The observed patterns emphasize the importance of considering specific ingredients when assessing the microbiological safety of pet food formulations.

The microbial quality of pet food samples significantly varies by the country of origin (*p*-value = 0.002). Pet foods sourced from the United States demonstrate the highest percentage of samples (90.50%) conforming to acceptable TAMC levels, indicative of superior overall microbiological quality. Conversely, pet foods from Hungary display a higher percentage of samples (61.10%) with unacceptable TAMC levels. These disparities underscore the importance of considering global variations in manufacturing practices, storage conditions, and ingredient sourcing. International collaboration and standardization are crucial to ensure consistent, high-quality pet food products worldwide.

### 3.2 *Enterobacteriaceae* spp.

In accordance with EU regulations No 142/2011 (19), pet food samples, excluding canned ones, containing dog chews and processed products that surpass the threshold of 3 × 10^2^ CFU/g for *Enterobacteriaceae* are deemed unsatisfactory in terms of microbial hygiene.

Presumptive *Enterobacteriaceae* was identified in 12 out of the 118 samples examined, constituting around 10% of the total. Among the 12 positive samples, 8 (7%) surpassed the limit, indicating elevated contamination. The *Enterobacteriaceae* levels in our investigation ranged from below the quantification limit (BQL) to more than 3 × 10^7^ CFU/g.

Our results reveal lower levels of *Enterobacteriaceae* contamination compared to previous studies, such as the one conducted by Kazimierska et al. (35). In their study, *Enterobacteriaceae* were identified in six samples of dog food (30%) after 24 h of incubation, simulating conditions typical of home storage. Additionally, in the study by Wojdat et al. (36), it was reported that 10% of dry pet food samples in Poland exceeded the contamination level of 3 × 10^2^ CFU/g. Additionally, 30% of the pet food samples from Lebanon were contaminated with *Enterobacteriaceae* (16). Conversely, Holda et al. (12) reported a higher contamination rate of 60% in dog food samples in Poland. Hellgren et al. (37) demonstrated pathogenic bacteria presence in raw pet food, with 60% exceeding maximum levels. Another study in Switzerland revealed that 72.5% of raw pet food samples did not meet EU microbiological regulations (38). Raw food’s elevated contamination, attributed to the lack of heating and processing, was evident in the screening for extended-spectrum beta-lactamase-producing *Enterobacteriaceae*, isolated from 77.8% of raw pet food and 0% from non-raw pet food (39).

It is important to take into account the methodological disparities between our study and prior research, as these could be influential in explaining discrepancies in outcomes. An evident contrast lies in the sampling methodologies utilized across studies. For example, Kazimierska et al. (35) simulated scenarios typical of home storage conditions, potentially resulting in elevated contamination rates due to prolonged exposure to environmental factors. Variations in microbial detection methods and pet food processing procedures like incubation periods, culture media, and identification protocols may lead to varying outcomes regarding *Enterobacteriaceae* presence and quantity. Moreover, discrepancies in pet food processing, such as heating duration and environmental conditions, can affect microbial survival and proliferation. These factors highlight the need for standardized methodologies and consideration of environmental variables in assessing contamination levels in pet food.

Carvalho et al. (40) emphasized that pets, particularly dogs, are significant sources of multiresistant *Escherichia coli* strains in households, posing health risks through various transmission routes. Takahashi et al. (41) noted that food manufacturers consider *Enterobacteriaceae* an indicator of hygiene, implying poor sanitation or improper processing in pet food production. Certain *Enterobacteriaceae*, *like E. coli* and *Enterobacter* spp., can lead to extraintestinal opportunistic infections in dogs, including urogenital infections, meningitis, sepsis, and surgical site infections (42). This underscores the importance of monitoring and improving hygiene practices in the pet food processing environment to mitigate health risks for both animals and humans.

In our examination of *Enterobacteriaceae* contamination across various pet food categories, we found that dry pet food exhibits a higher contamination rate compared to canned food. However, this difference lacks statistical significance (*p*-value = 0.079). Our results align with Serhan et al.’s (16), who found higher contamination with *Enterobacteriaceae* contamination in dry samples, likely due to prolonged survival in low-moisture conditions (43). Similar trends in infant formula production suggest risks during the dry phase (44). However, our findings contradict studies by Kukier et al. (18) and Kepińska-Pacelik et al. (20), which indicated a higher level of *Enterobacteriaceae* contamination in canned foods compared to dry foods.

Dogs demonstrate slightly higher levels of *Enterobacteriaceae* contamination compared to cats. Among adults, there is a higher contamination rate than among puppies or kittens, the latter consistently displaying a 100% acceptance rate, indicating no contamination above the limit. Statistical analysis yields *p*-values of 0.664 and 0.999, revealing no significant difference between pet type and age, respectively. Our findings are consistent with Kazimierska et al. (35), whose analyses similarly do not support the claim that canned dog food formulated for various age groups exhibits differences in microbiological purity.

Our study revealed that the inclusion of grains in pet food does not significantly affect contamination levels. This finding aligns with previous research examining the microbiological quality of canned foods for both puppies and adult dogs, which found no confirmed association between the inclusion of a grain component in canned dog food and an increased risk of microbiological contamination by *Enterobacteriaceae* (35). However, it is essential to highlight that our results contrast with those found in pet food samples obtained from Lebanon. In this context, dry pet food containing grains showed increased contamination of *Enterobacteriaceae* compared to grain-free alternatives, as indicated by the study conducted by Serhan et al. (16).

Across various food sources and countries of origin, the majority of samples meet acceptable criteria, highlighting the overall safety of pet food. It is important to note that microbiological standards, which specify permissible microorganism levels in pet food, are enforced in the UAE. This stands in contrast to Lebanon, where Asian countries demonstrated an approximately 26% rate of unacceptable *Enterobacteriaceae* contamination (16).

### 3.3 Total yeasts and molds count

Cereals, a crucial component in pet food, have the potential to serve as carriers for harmful mycotoxins produced by molds, posing a health risk to both pets and their owners (45). The permissible limit for the total count of yeasts and molds should not exceed 10^4^ CFU/g (22). In the present investigation, contamination levels varied from 0 to 3 × 10^7^ CFU/g. Out of the samples, 33 (27%) surpassed the established contamination limit.

Our findings differ from those presented by Kazimerska (35) where no TYMC contamination was identified in dog pet foods samples, and by Holda et al. (12), who found no fungal contamination exceeding 2 × 10^2^ CFU/g. The sample sizes across studies vary significantly, with Kazimierska investigation comprising 20 samples of commercial canned dog foods, while Holda’s study involved four complete dry foods tailored for growing dogs. In contrast, our research encompasses a notably larger sample size of 118 pet food samples. This considerable difference in sample size might have implications for the observed levels of yeast and mold contamination since smaller sample sizes may not capture the full spectrum of the bacterial contamination. It is plausible that the variation in sample size could contribute to the comparatively lower contamination levels identified in our study, highlighting the importance of sample size considerations in microbial contamination research.

Moreover, our results surpass those of several studies. Serhan et al. (16) reported that 12% of the samples exhibited contamination levels surpassing 10^4^ CFU/g, while Wojdat et al. (36), found a 9% contamination level above 10^4^ CFU/g. Additionally, Kazimierska et al. (28) found that molds, potentially accountable for the presence of mycotoxins, were detected in approximately 20% of the examined dog food samples. Furthermore, in their assessment of pet food’s microbiological quality Bueno et al. (46) observed that all tested commercial dry dog food samples were contaminated with yeasts and molds.

Certain molds and certain yeasts found in food can be harmful to animals due to their capacity to generate mycotoxins. The introduction of these toxins can occur through various pathways. Factors influencing mycotoxin production include weather, presence of inhibitors and nutrients, geographical and seasonal variations, humidity, temperature, crop susceptibility, cultivation methods, harvesting practices, as well as storage and transportation procedures (47). Understanding these factors is crucial for managing mycotoxin risk. For instance, weather and seasonal changes affect pre-harvest mold development, while humidity and temperature during storage/transportation impact post-harvest contamination. Cultivation methods and harvesting practices also influence crop vulnerability to mold infestations.

Dry samples show a slightly higher TYMC contamination rate compared to canned samples, but the computed *p*-value of 0.479 suggests that there is no statistically significant difference, suggesting comparable microbiological quality for both types. In 2014, research conducted by Blajet-Kosicka et al. (15) revealed that various molds, including *Aspergillus*, *Mucor*, and *Penicillium*, as well as a range of mycotoxins, were identified in dehydrated pet food.

Analyzing both pet type and age reveals insights into microbial contamination levels. Dogs exhibit a slightly higher percentage of samples within acceptable TYMC limits compared to cats. Similarly, adult pet food samples demonstrate a slightly higher percentage within acceptable TYMC limits compared to samples for puppies or kittens. The *p*-values of 0.717 and 0.445 for pet type and pet age, respectively, indicate no significant association between them and TYMC levels. These findings underscore the importance of considering nutritional requirements, ingredient preferences, and formulation processes specific to each pet species and age group, as these factors could impact microbial contamination patterns.

The calculated p-value of 0.971 for grain and grain-free pet food suggests no significant difference in microbial contamination based on the presence of grains. This contrasts with previous studies, noting the correlation observed between mold presence and cereals in dog food. Specifically, yeast and mold were more frequently detected in dog foods containing grains, with six out of seven positive results corresponding to grain-included foods, accounting for 86% of the cases (28). Analyzing pet food samples from Lebanon, no significant correlation was observed between grain presence and TYMC levels. However, it was noted that all eight samples (12%) exceeding the TYMC limit contained at least one cereal (maize, rice, wheat, and/or oats) (16). Although our study did not find a significant link between the presence of grains and TYMC levels in pet food, further investigation into this discrepancy is warranted. One possible reason could be the diversity in processing methods utilized by pet food manufacturers. Varied processing methods utilized by pet food manufacturers may impact the susceptibility of grains to mold contamination and subsequent mycotoxin production. Additionally, regional disparities in agricultural practices and grain procurement methods could contribute to fluctuations in microbial contamination levels among different batches of pet food.

An analysis of various food sources including poultry, fish, beef, liver, and turkey reveals high percentages of samples within acceptable TYMC limits, indicating good overall microbiological quality. Most *p*-values for various food sources are above 0.05, suggesting no significant association between specific food sources and TYMC levels. However, variations in the percentage of samples within acceptable TYMC limits are observed among pet food from different countries, although the associated p-value of 0.144 suggests a potential association between the country of origin and TYMC levels, it does not reach statistical significance. These findings highlight the importance of exploring specific ingredients, processing methods, and geographical sources to identify potential sources of contamination and ensure consistent microbiological quality across different pet food products.

### 3.4 *Salmonella* spp.

Exploring the prevalence of *Salmonella* in pet food reveals a multifaceted landscape, highlighting global implications for pet food safety. The absence of *Salmonella* in our tested samples aligns with stringent EU regulations (19) and corroborates findings from D’aoust et al. (48) and Kazimierska (35), providing a reassuring outlook on the safety of pet food in some regions. A study conducted in Poland revealed that only 0.96% of dry pet food products and none of the canned ones tested positive for *Salmonella* (49). This contrasts with a broader examination of microbial prevalence in pet food, indicating an 8% positive rate for *Salmonella* species, with all positive cases attributed to raw feed and only 1 case found in dry pet food (14). In Sweden, Hellgren et al. (37) reported a 7% contamination rate in tested raw meat-based products, while Yukawa et al. (50) observed a 2% incidence of *Salmonella* in dog treats collected in Japan. However, our results stand in contrast to a study conducted in Lebanon, where *Salmonella* was detected in 41% of the total pet food samples (16). These findings underscore the variability in *Salmonella* prevalence in pet food across different geographical locations, reinforcing the importance of regional variations.

To thoroughly assess *Salmonella* prevalence in pet food, we must examine the methodologies used across studies. While many employ culture-based methods, some variations exist, potentially impacting sensitivity levels compared to molecular techniques like PCR, as employed by Yukawa et al. (50). Variations in sample sizes and methodologies may introduce bias; for instance, some studies tested fewer than 100 samples (35, 37, 48), while others examined over 1,000 (14, 49). These differences, alongside variations in sample collection and storage, may affect result reliability. It is crucial to note that each study was conducted in specific regions, underscoring the importance of acknowledging inherent limitations such as potential selection bias and inability to capture temporal fluctuations in *Salmonella* prevalence.

An alarming instance in the United States involved an outbreak of *Salmonella Schwarzengrund* associated with dry dog and cat food from a specific manufacturer, leading to 79 identified cases (13). Notably, no *Salmonella* species were found in any of the pet food samples from the UAE, mitigating the potential transmission of zoonotic pathogens, such as *Salmonella*, from pets to humans. While the likelihood of transmission from pets to humans is generally rare, it is essential to acknowledge that some pets may carry diseases asymptomatically. Dogs and cats, even without apparent symptoms, can act as carriers for extended durations, potentially infecting individuals who handle contaminated pet food or have direct contact with these animals. Importantly, asymptomatic pets can shed *Salmonella* for up to 3 months (51), underscoring the necessity for ongoing vigilance and adherence to safety measures when handling pet food.

Enforcing stringent safety protocols during pet food handling and production, including thorough sanitation, pathogen screening, and regulatory adherence, is vital for averting *Salmonella* contamination. Educating pet owners about potential hazards and encouraging careful feeding practices further reduces the risk of transmission between animals and humans.

### 3.5 *Listeria monocytogenes* spp.

In relation to *L. monocytogenes*, its presence should be either undetectable in 25 g of pet food or its contamination level must be less than 100 CFU/g (21). In our investigation, *L. monocytogenes* species were identified in 44 out of 118 samples, accounting for 37%. This contamination level was lower than that documented for pet food in Lebanon, where it reached 64% (16), but higher than a previous study by Nemser et al. (14), which revealed 16% positive samples for *L. monocytogenes monocytogenes* and 14% for another *L. monocytogenes* spp. Additionally, none of the 20 analyzed canned pet foods in Poland showed any evidence of *L. monocytogenes* contamination (35).

As outlined by the Center for Veterinary Medicine (17), *L. monocytogenes* spp. can elicit mild gastrointestinal symptoms, fever, muscle pain, breathing difficulties, pregnancy loss, and even fatality. Pet owners should be aware that, although certain cats and dogs may not exhibit signs of Listeriosis following the consumption of contaminated pet food, they can still serve as carriers of *L. monocytogenes monocytogenes*, excreting it in their stool and subsequently spreading it within the household. Given the close relationship between pets and their owners, understanding and addressing *L. monocytogenes* contamination in pet food is crucial.

The data reveals a significant disparity in *L. monocytogenes* prevalence between dry and canned pet food. Dry pet food exhibits a higher positive rate compared to canned pet food. Nevertheless, the *p*-value of 0.673 suggests that this distinction lacks statistical significance, implying that the type of pet food may not be a major contributor to *L. monocytogenes* presence. A study conducted by Mireille et al. (16) identified a notable difference between dry and canned foods, with dry foods showing a higher *L. monocytogenes* contamination rate than canned ones.

When examining the type of pet, the data indicates that cats exhibit a higher positive rate for *L. monocytogenes* compared to dogs. The *p*-value of 0.019 suggests a significant disparity in *L. monocytogenes* prevalence between cats and dogs, with cats appearing more susceptible. Additionally, Lebanese pet food products for dogs and cats showed a significant distinction in *L. monocytogenes monocytogenes* prevalence (16). When discussing the higher prevalence of *L. monocytogenes* in cats compared to dogs, several factors should be considered. One possible explanation is the dietary differences between the two species. Cats’ more acidic digestive systems compared to dogs’ may affect their susceptibility to *L. monocytogenes* infection. Conversely, dogs being omnivores, can tolerate a wider range of foods, including plant-based ingredients (52), potentially resulting in lower *L. monocytogenes* contamination in their food. Differences in feeding behaviors, such as scavenging habits and food preferences, could also contribute to the observed disparities. Moreover, cats, especially those allowed outdoors, may have increased exposure to potential sources of *L. monocytogenes* contamination like rodents or contaminated soil, while indoor cats may also be exposed to *L. monocytogenes* through contaminated pet food or contact with humans who handle raw meat. Conversely, dogs, especially with wildlife may have fewer opportunities for exposure to *L. monocytogenes* in their environment.

Age appears to influence *L. monocytogenes* prevalence, with adult pets demonstrating a lower positive rate compared to puppies or kittens. Although the *p*-value of 0.073 is not highly significant, it hints at a potential trend toward higher *L. monocytogenes* presence in puppies or kittens.

Contrary to findings by Mireille et al. (16), the presence or absence of grains in pet food does not significantly impact *L. monocytogenes* prevalence in our study. Their research indicated higher *L. monocytogenes* contamination in grain foods.

Table 5 dissects *L. monocytogenes* prevalence based on various food sources, revealing notable differences. Higher prevalence is noted when beef is present, whereas lower prevalence is associated with the presence of turkey. However, the varying *p*-values for these categories suggest that the impact of food source on *L. monocytogenes* presence may be specific to certain types of meat.

The data offers insights into the geographical variation of *L. monocytogenes* prevalence in pet food. Although differences exist between countries, the p-value of 0.129 indicates that the observed variations may not attain statistical significance.

### 3.6 *Clostridium* spp.

The packaging conditions of pet food, whether aerobic for dry food or anaerobic for canned food, can have significant implications for the survival and potential growth of Clostridium species. In dry pet food, aerobic packaging conditions inhibit the growth of Clostridium, while in canned pet food, anaerobic packaging conditions may allow Clostridium spores to persist and pose a risk if proper handling and storage practices are not followed. Our study revealed *Clostridium* species in 28 (24%) out of 118 samples, with bacterial loads ranging from 0 to 3,680 CFU/g. In contrast to a Polish study that reported no *Clostridium* contamination in pet foods (35), our findings align with Hellgren et al.’s (37) research, which reported a 30% contamination rate, particularly with *C. perfringens*. Similarly, Wojdat et al. (53) found *Clostridium perfringens* in 38% of analyzed animal feeding stuffs, influenced by factors such as feed material quality and storage conditions. Other studies focused on the presence of *Clostridium difficile* in dogs and their households, revealing contamination rates of 10% in dogs and 31% in households. Additionally, a commercial pet food containing turkey as the main ingredient was found to be contaminated with *C. difficile* (54, 55).

Recent research has discovered *Clostridium* species in dogs, contributing to both acute and chronic diarrhea. These bacteria, found widely in dog feces, share similarities with those causing diseases in humans (56, 57). Moreover, they are present in various food sources, including meat, poultry, pork, and beef products, particularly in those prepared with sauce (58). That is why, it is crucial to examine the health patterns of pets exposed to contaminated food. Observing whether there are identifiable connections between *Clostridium* contamination and issues like gastrointestinal problems in pets can provide valuable insights. Furthermore, comprehending the effects on the general well-being of animals not only informs veterinary practices but also contributes to the formulation of preventive measures.

According to Table 7, dry pet food exhibits a contamination rate of 33.30%, slightly higher than canned pet food’s rate of 20.90%. Similarly, while dogs show a slightly higher contamination rate than cats, and puppies or kittens show a slightly higher rate compared to adults, the respective *p*-values (pet food type: 0.182; pet type:0.215; pet age: 0.390) indicate no statistical significance. These findings suggest that neither the sample type, pet type or pet age significantly influence *Clostridium* contamination.

Moreover, the presence or absence of grains in pet food does not significantly impact *Clostridium* prevalence. Table 7 provides insights into *Clostridium* prevalence based on various food sources, revealing notable differences with variations in contamination rates based on specific ingredients. However, the *p*-values for these categories vary, suggesting that the impact of food source on *Clostridium* presence may be specific to certain types of meat.

The country of origin significantly impacts *Clostridium* contamination. Remarkably, pet foods from France show a 0% contamination rate, while those from the United States exhibit a lower contamination rate (14.30%) compared to others. The p-value of 0.029 indicates that the observed variations in contamination rates between countries may be statistically significant. The substantial influence of the pet food’s country of origin on *Clostridium* contamination rates indicates a complex interaction involving regulatory standards, production practices, and ingredient procurement. Countries with strict regulations and effective quality control procedures may experience lower contamination rates, while those with lax enforcement measures could encounter more difficulties in ensuring product safety. Cultural choices, ingredient compositions, and environmental conditions are additional factors likely affecting these discrepancies. Further investigation is necessary to comprehend the precise mechanisms driving these variations and devise tailored strategies to mitigate microbial risks in pet food worldwide.

In the context of *Clostridium* contamination in pet food, a crucial concern is the potential emergence of antibiotic resistance, particularly in strains like *Clostridium difficile*. Known for their adaptability through genetic changes, *C. difficile* has a versatile genome with mobile elements encoding antibiotic resistances (59–61). The presence of antibiotic-resistant strains in pet food poses challenges in veterinary medicine and public health. The transfer of antibiotic resistance between pets and humans is a growing concern. Studying the antibiotic susceptibility of *Clostridium* isolates in pet food can unveil patterns of resistance, providing insights into broader implications for both animal and human health.

## 4 Conclusion

Our comprehensive analysis of pet food SKUs marketed in UAE underscores the crucial importance of implementing robust quality control measures within the industry. The prevalence of microbial contamination, particularly the exceeding of acceptable limits in Total Aerobic Microbial Count (TAMC) and Total Yeast and Mold Count (TYMC) in 33 samples (28%), along with the presence of potentially harmful bacteria like *Enterobacteriaceae* in 8 samples (7%), *L. monocytogenes* in 44 samples (37%), and *Clostridium* in 28 samples (24%), highlights significant health risks associated with pet food consumption.

Importantly, our research addresses a critical gap, providing insights into pet food safety in the United Arab Emirates (UAE), emphasizing the necessity for tailored safety measures in this dynamic market. As the UAE plays a global role, our findings contribute to ongoing efforts for the safety and well-being of pets, guiding future research and industry practices. This research serves as a foundation for promoting global cooperation and enhancing the international standards for pet food safety.

## Data availability statement

The raw data supporting the conclusions of this article will be made available by the authors, without undue reservation.

## Author contributions

MH: Data curation, Formal analysis, Methodology, Validation, Writing – original draft. NA: Conceptualization, Funding acquisition, Investigation, Resources, Writing – original draft. HD: Formal analysis, Methodology, Writing – original draft. MD: Data curation, Methodology, Writing – original draft. SK: Resources, Writing – original draft. MS: Project administration, Resources, Supervision, Writing – original draft. HH: Conceptualization, Formal analysis, Funding acquisition, Investigation, Project administration, Resources, Supervision, Validation, Writing – original draft.
